# Simultaneous improvement in production of microalgal biodiesel and high-value alpha-linolenic acid by a single regulator acetylcholine

**DOI:** 10.1186/s13068-015-0196-0

**Published:** 2015-01-29

**Authors:** Ali Parsaeimehr, Zhilan Sun, Xiao Dou, Yi-Feng Chen

**Affiliations:** Laboratory of Biosystems Engineering, Institute of Biotechnology, Jiangsu Academy of Agricultural Sciences, Nanjing, 210014 Jiangsu China; Targetong Energy Co., Ltd, Nanjing, 211800 Jiangsu China

**Keywords:** Acetylcholine, *Chlorella sorokiniana* U2-9, Lipids, Alpha-linolenic acid, Biodiesel

## Abstract

**Background:**

Photoautotrophic microalgae are a promising avenue for sustained biodiesel production, but are compromised by low yields of biomass and lipids at present. We are developing a chemical approach to improve microalgal accumulation of feedstock lipids as well as high-value alpha-linolenic acid which in turn might provide a driving force for biodiesel production.

**Results:**

We demonstrate the effectiveness of the small bioactive molecule “acetylcholine” on accumulation of biomass, total lipids, and alpha-linolenic acid in *Chlorella sorokiniana*. The effectiveness exists in different species of *Chlorella*. Moreover, the precursor and analogs of acetylcholine display increased effectiveness at higher applied doses, with maximal increases by 126, 80, and 60% over controls for biomass, total lipids, and alpha-linolenic acid, respectively. Production of calculated biodiesel was also improved by the precursor and analogs of acetylcholine. The biodiesel quality affected by changes in microalgal fatty acid composition was addressed.

**Conclusion:**

The chemical approach described here could improve the lipid yield and biodiesel production of photoautotrophic microalgae if combined with current genetic approaches.

**Electronic supplementary material:**

The online version of this article (doi:10.1186/s13068-015-0196-0) contains supplementary material, which is available to authorized users.

## Background

Energy consumption is dramatically increasing, and the global energy demand is estimated to grow by more than 85% by 2040. Fossil fuel resources provide most of the world's energy demands but are limited, and thus additional sources of energy must be considered. Biofuels have the potential to supply a portion of our society’s energy demands. Biodiesel is a diesel fuel derived from animal or plant oils and is composed of methyl esters of long-chain fatty acids produced by transesterification of lipids [1].

Microalgae have the capacity to generate considerable amounts of biomass and lipids, which can be used for biodiesel production and are also of potential use for human health [2,3]. Microalgae offer excellent sources of polyunsaturated fatty acids (PUFAs), including docosahexaenoic acid (DHA, C22:6), eicosapentaenoic acid (EPA, C20:5), arachidonic acid (ARA, C20:4) and alpha-linolenic acid (ALA, C18:3) [4,5]. These essential fatty acids are important to human health and must be consumed regularly, since they cannot be synthesized by humans [6-8].

A recent pharmacological survey suggested that small bioactive molecules might be effective in microalgal lipid accumulation [9]. We examined the feasibility of this approach using the small bioactive molecule acetylcholine (ACh). ACh is an organic compound and is probably one of the most evolutionarily ancient signaling molecules. ACh is best known as a canonical neurotransmitter and plays a fundamental function in the neurotransmission process in animals and insects [10,11]. However, ACh is also likely to play a role in non-neuronal signaling, as it is found in a range of organisms including plants, algae, fungi, protozoa, and bacteria. In plants, ACh exhibits stimulatory roles in flowering, stomatal movements, and phytochrome action, as well as inhibitory roles in ethylene production and leaf rolling [12-15]. ACh is also reported to stimulate growth in *Vigna sesquipedalis*, *Raphanus sativus, Phyllostachys bambusoides, Triticum aestivum*, and *Lycopersicon esculentum* [16-19]. Light has a regulatory role in ACh production, and ACh plays the roles of cholinergic agonist and antagonist in the growth and differentiation in the green alga *Micrasterias denticulata* [20]. Choline and acetyl coenzyme-A are responsible for ACh synthesis mediated by the enzyme choline acetyltransferase (ChAT) (Choline + Acetyl coenzyme A ⇌ Acetylcoline + Coenzyme A). Since the rate of ACh biosynthesis is correlated to the presence of ACh precursor and associated enzymes, ACh can influence the biosynthesis process by regulating enzymes such as ChAT, acetyl-CoA carboxylase, choline kinase, cholinesterase, and pseudocholinesterase [21,22]. Reports have pointed to the existence of ACh in 0.2 μg g^-1^ of dry weight in microalgae species such as *Micrasterias denticulata* and *Laurencia obtusa*, even though ACh was only detectable when extracts were pre-treated by ACh-esterase inhibitor [20,23]. ACh and its chemical analog taurine had significant stimulations on some metabolites of *Chlorella vulgaris,* although ACh had a superior activity on production of monosaccharides and soluble proteins [24]. In this study, we report that ACh improves the efficiency for accumulation of microalgal lipids and ALA, a valuable compound in *Chlorella sorokiniana* U2-9. The effectiveness of ACh significantly improved lipid production in a variety of microalgal species.

## Results and discussion

### *Chlorella sorokiniana* U2-9 dominantly performs photoautotrophic growth under light in TAP medium

In this study, Tris-Acetate-Phosphate (TAP) medium was used to culture *C. sorokiniana* U2-9*.* The inclusion of acetate in the medium might potentially support several metabolic pathways, including heterotrophy, photoautotrophy, or photomixotrophy. Therefore, two sets of experiments were performed to clarify that photoautotrophic growth is dominant in TAP medium. In the first set of experiments, biomass was much higher (2.5 ± 0.4 g L^-1^) in the light than in the dark (0.9 ± 0.3 g L^-1^), indicating that light is an undeniable factor for growth of *Chlorella*. In the second experiment, an obvious stimulation of biomass was observed by a higher dose of CO_2_ (for example, 3.8 ± 0.5 g L^-1^ at 5% CO_2_) compared to the culture with air aeration (2.5 ± 0.4 g L^-1^), indicative of CO_2_ dependence of *Chlorella* growth. Taken together, this means that TAP medium mostly supports the growth of *Chlorella* by photosynthesis, and covers a relatively simple mode of metabolism which could be suitable for the evaluation of ACh effects.

### Multiple effects of acetylcholine on *Chlorella sorokiniana* U2-9 and derived biodiesel

The comprehensive positive effects of ACh on growth, lipid content, and lipid profiling of the *Chlorella* species were examined and revealed. An increase in dry weight was observed with 5 and 10 μg L^-1^ doses of ACh at the initial growth phase of *C. sorokiniana* U2-9, and the dry weight was increased by 26.38 ± 2% in comparison to the control. Nevertheless, the usage of ACh at the exponential phase (day 4, OD_680_ = 1.62 ± 0.15) of algal growth was more effective, and higher dry weight (3.2 ± 0.15 g L^-1^) was obtained with a 10 μg L^-1^ dosage of ACh. Using different doses of ACh at the stationary phase of *C. sorokiniana* growth (day 9, OD_680_ = 2.82 ± 0.1) had no effect on algal growth; however, higher applied doses of ACh (100 μg L^-1^) showed a rapid and negative effect, similar to an algicide.

The lipid content improved from 214.3 ± 14 mg g^-1^ of dry weight to 303.3 ± 27 mg g^-1^ of dry weight (lipid productivity of 80 ± 6 mg L^-1^ day^-1^) using a 5 μg L^-1^ dose of ACh at the initial phase of *C. sorokiniana* growth, but was decreased to 190 ± 10 mg g^-1^ of dry weight with a 10 μg L^-1^ dosage of ACh. ACh supplied at the exponential growth phase of *C. sorokiniana* demonstrated a greater influence on lipid production, and the total lipid content was increased up to 312 ± 54 mg g^-1^ of dry weight with a 5 μg L^-1^ dosage of ACh (lipid productivity of 92 ± 10 mg L^-1^ day^-1^, and an increase by 45.8%). Nevertheless, with a 10 μg L^-1^ dosage of ACh the total lipid content and lipid productivity were reduced by 23.9 and 59.6% (total lipid content: 163.3 ± 20.8 mg g ^-1^ of dry weight, lipid productivity_:_ 52 ± 8 mg L^-1^ day^-1^) . These results revealed that the ACh stimulation on the microalgal growth and lipid accumulation is growth phase- and dose-dependent.

(See Table 1 and Additional file 1: Table S-1).

**Table 1:** **Effectiveness of acetylcholine on** ***Chlorella sorokiniana*** **U2-9**

	**ACh** **(μg L** ^**−1**^ **)**						
	**0**	**0.125**	**0.25**	**0.5**	**1**	**5**	**10**
Dry weight (gL^−1^)	2.16 ± 0.2	2.2 ± 2	2.2 ± 0.2	2.4 ± 0.17	2.43 ± 0.2	2.73 ± 0.1	2.66 ± 0.15
Total lipid content (mg g^−1^ of dry weight)	214.3 ± 14	223.3 ± 16	231.6 ± 12	252 ± 10	296 ± 26	303 ± 24	190 ± 12
Lipid productivity (mgL^−1^ day^−1^)	46.4 ± 6	49.3 ± 7	50.8 ± 4	60.5 ± 4	72.4 ± 8	82.8 ± 6	50.7 ± 5

**Note:** Acetylcholine was more efficient at doses of 0.5 to 5 μg L^−1^, but it exposed negative effects on lipid content and lipid productivity at 10 μg L^−1^. ACh was added at the initial phase of algal growth. All data were given as mean ± standard error (n = 3) of three separated tests.

The GC analysis showed that the major components of fatty acids in *C. sorokiniana* U2-9 were palmitic acid (C16:0), stearic acid (C18:0), oleic acid (C18:1), linoleic acid (C18:2), and ALA (C18:3). During the growth of microalgae these components were altered slightly; for example, ALA (C18:3) was increased from 14.7 to 18.7%. However, the treatment of microalgae with ACh significantly improved the relative content of ALA. ACh supplied in the initial phase of *C. sorokiniana* growth enhanced the relative content of ALA by 61.2%, resulting in an increase of the PUFAs up to 50% of the fatty acids profile, and also in an increase of the biodiesel yield from 17.7 ± 6% to 30.9 ± 7% over the controls. Similar effects of ACh were also generated when ACh was supplied in the stationary phase of microalgal growth (Additional file 1: Table S-2).

**Figure 1:**
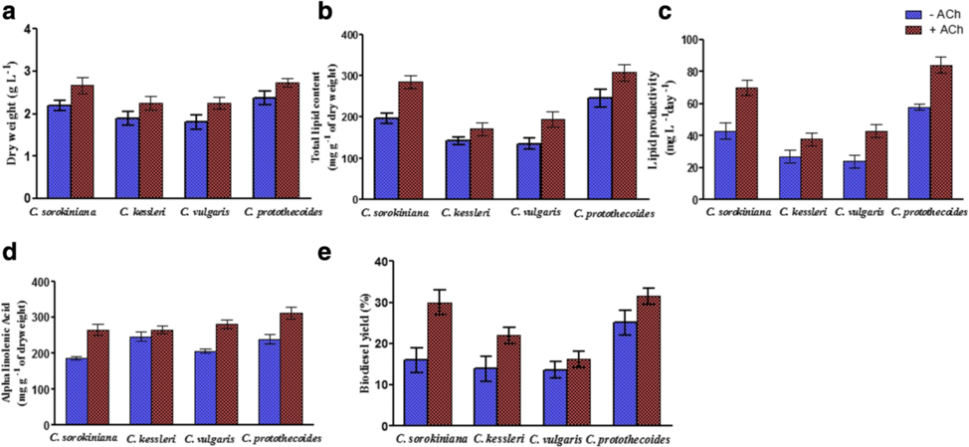
**Effectiveness of ACh on other species of the** ***Chlorella*** **genus. (a-c)** The dry weight, lipid content, and lipid productivity of other *Chlorella* species were stimulated by ACh. **(d)** A higher ALA content was determined at 311.3 ± 17 mg g^-1^ of dry weight in *C. Protothecoides* UTEX 256 treated with ACh. **(e)** ACh was efficient in increasing the biodiesel yield to 31.5 ± 2% in ACh-treated samples of *C. Protothecoides* UTEX 256. ACh was used at the initial phase of algal growth at a dosage of 5 μg L^-1^ in TAP medium. All data were expressed as mean ± standard error (n = 3) of three separate tests.

**Figure 2:**
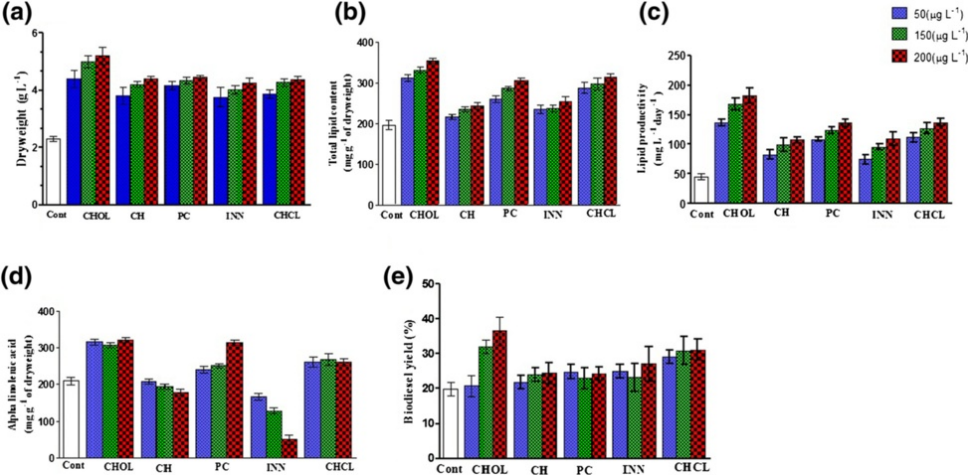
**The influences of ACh precursor and analogs on** ***C. sorokiniana*** **U2-9. (a-c)** Choline was more efficient for the dry weight, lipid content, and lipid productivity in comparison with ACh analogs. **(d)** The production of ALA was improved up to 321 ± 7 and 315 ± 5 mg g^-1^ of dry weight by choline and phosphatidylcholine, respectively. **(e)** The biodiesel yield was improved up to 36.5 ± 4% by 200 μg L^-1^ choline. Note: CHOL: choline, CHCL: choline chloride, CH: choline hydroxide, INN: citicoline, and PC: phosphatidylcholine. Samples with no drug treatments were used as control (Cont). All data were expressed as mean ± standard error (n = 3) of three separate tests.

Since the ratio of saturated to unsaturated fatty acids of the lipid profiling determines the quality of microalgal biodiesel, and since the relative contents of the microalgal fatty acid profile were altered by ACh, we calculated the biodiesel properties and estimated ACh influences on the iodine value (IV), the cold filter plugging point (CFPP), and the cetane number (CN); these parameters are closely associated with the biodiesel quality. The CN of biodiesel is linked to the ignition quality; a shorter ignition time is associated with an increase of the CN. The IV shows the biodiesel vulnerability to oxidative attacks and is connected to numbers and positions of double bonds in the carbon chains of alkyl esters. The CFPP indicates the flow performance of biodiesel at low temperatures and is linked to the amounts of unsaturated fatty acids in biodiesel [25]. In general, we observed that the IV was enhanced and the CFPP was reduced in biodiesels from the ACh-treated samples, implying an improvement in the stability of biodiesel (Additional file 1: Table S-2). In contrast, the CN was almost not affected by the ACh treatments. If the ALA was removed from the fatty acid profile, the CN of the derived biodiesel could be greatly increased (Figure 1e, Figure 2e, Table 2).

**Table 2:** **Estimation of biodiesel properties based on fatty acid profiles in included and excluded ALA treatments**

		**SV**	**IV**	**DU**	**LCSF**	**CFPP(°C)**	**CN**
Included ALA	Control	210.9	133.8	122.9	2.6	−8.1	42.1
	ACh	211.1	149.1	128.6	2.4	−9.0	38.6
	CHOL	210.6	160.2	119.0	4.4	−2.5	36.1
	CH	211.43	114.45	56.59	6.6	4.28	46.36
	CHCL	210.8	162.5	99.5	3.6	−4.9	35.6
	PC	211.2	158.3	100.6	2.6	−8.1	36.5
	INN	211.6	100.1	57.7	7.3	6.5	49.5
Excluded ALA	Control	212.55	109.71	109.54	3.08	−8.15	47.29
	ACh	213.97	114.86	108.93	3.05	−6.89	45.96
	CHOL	214.95	109.12	82.4	6.4	3.7	47.2
	CH	213.72	73.81	56.59	8.3	9.59	55.22
	CHCL	213.08	122.12	63.32	4.94	−0.93	44.43
	PC	215.36	108.53	57.67	3.81	−4.5	47.22
	INN	212.7	67.68	31.24	8.66	10.73	56.73

Note: ACh: acetylcholine; CHOL: choline; CHCL: choline chloride; CH: choline hydroxide; INN: citicoline; PC: phosphatidylcholine. ALA: alpha-linolenic acid. CN: cetane number; SV: saponification; IV: iodine value; DU: degree of unsaturation; LCSF: long-chain saturation factor; CFPP: cold filter plugging point. No drug treatments were used as control. All data were expressed as the mean of three separate tests.

### Effectiveness of acetylcholine was confirmed stable at different levels of Tris acetate and phosphate buffer

**Figure 3:**
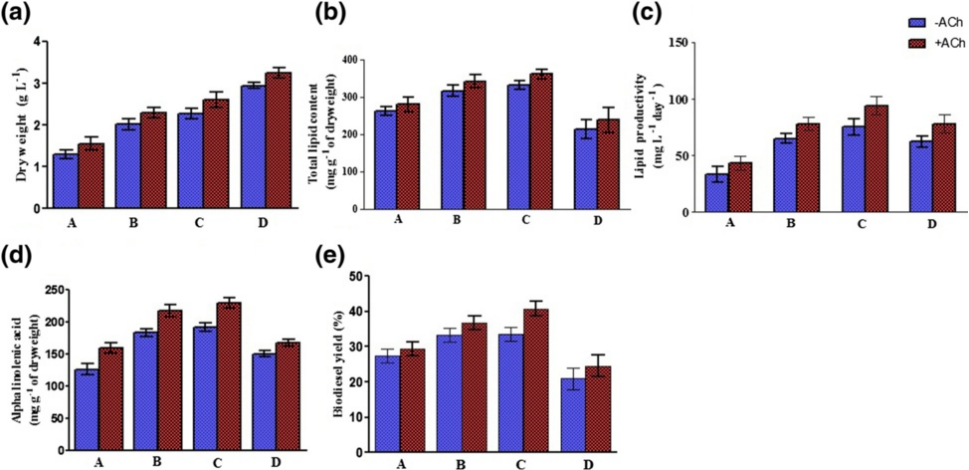
**Efficiency of ACh at dissimilar concentrations of Tris base and acetic acid.** **(a-c)** Acetylcholine (ACh) enhanced dry weight, lipid content, and lipid productivity of *C. sorokiniana* U2-9 at dissimilar concentrations of Tris acetate. **(d-e)** The highest ALA (228.74 mg g^-1^ of dry weight) and biodiesel yields (40.8%) were obtained at the level C of Tris acetate. Note: A-D describe the applied levels of Tris acetate. A: 1.2 g L^-1^ Tris base and 5 mL L^-1^glacial acetic acid, B: 2.4 g L^-1^ Tris base and 10 mL L^-1^ glacial acetic acid, C: 3.6 g L^-1^ Tris base and 12.5 mL L^-1^ glacial acetic acid, D: 4.8 g L^-1^ Tris base and 15 mL L^-1^ glacial acetic acid. All data were given as mean ± standard error (n = 3) of three separate tests.

**Figure 4:**
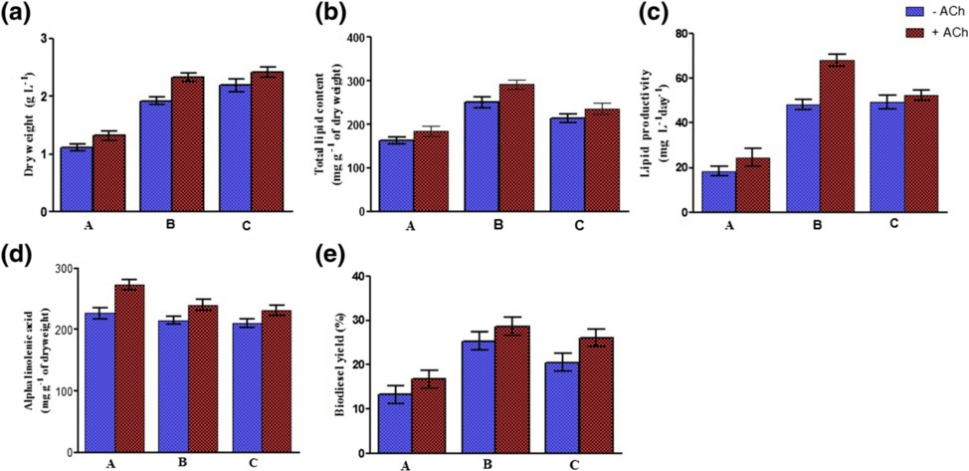
**Effectiveness of ACh at the altered levels of phosphate buffer. (a-c)** ACh improved the dry weight, lipid content, and lipid productivity of *C. sorokiniana* U2-9 at the altered phosphate buffer levels. **(d)** The highest ALA content was determined to be 272.43 ± 8 mg g^-1^ of dry weight at level A of phosphate buffer + ACh. **(e)** Biodiesel yield was increased up to 28.8 ± 2% at level B of phosphate buffer + ACh. Note: A-C describe the applied levels of phosphate buffer. A: Na_2_HPO_4_: 5.8 g L^-1^, KH_2_PO_4_: 3.63 g L^-1^, B: Na_2_HPO_4_: 11.62 g L^-1^ KH_2_PO_4_: 7.26 g L^-1^, C: Na_2_HPO_4_: 17.42 g L^-1^, KH_2_PO_4_: 10.89 g L^-1^. All data were given as mean ± standard error (n = 3) of three separate tests.

We further examined the stability of the ACh effects under varied culture conditions. For this purpose, different levels of Tris acetate and phosphate buffer were used to investigate whether the influence of ACh is stable on *C. sorokiniana* U2-9 through culture. Increases in concentrations of the Tris acetate and phosphate buffer improved the biomass yields, although the total lipid content, the lipid productivity, and the ALA yield were reduced or not changed at the fourfold dose of Tris acetate or the threefold dose of phosphate buffer. Under the complex situation, ACh could still further enhance the dry weight, the lipid content, the lipid productivity, the ALA level, and the biodiesel yield (Figures 3 and 4), supporting a consistent role of ACh. Interestingly, the biodiesel properties were similar to previous results (Additional file 1: Table S-2); that is, the IV was increased and the CFPP was decreased in the ACh-treated samples with increased levels of Tris acetate and phosphate buffer (Additional file 1: Tables S-3 and S-4).

### Pharmacological examination implied a role of an endogenous ACh

**Figure 5:**
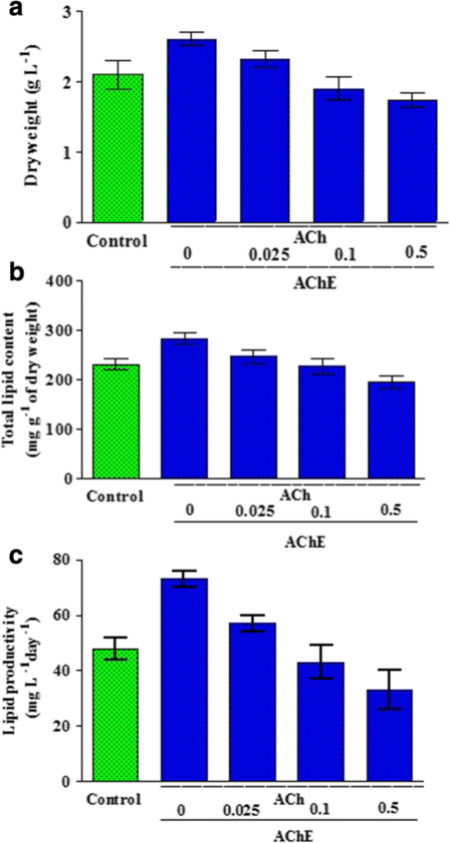
**ACh influence on** ***C. sorokiniana*** **U2-9 was inhibited by using acetylcholinesterase as an inhibitor.** **(a-c)** ACh influence on *C. sorokiniana* U2-9 was inhibited by using acetylcholinesterase as an inhibitor. The dry weight and lipid content in ACh-treated *Chlorella* cells were decreased by adding AChE in a range from 0.025 to 0.5 μg L^-1^. All data were given as mean ± standard error (n = 3) of three separate tests.

The amount of 8.6 ± 0.24 μg g ^-1^ of dry weight of ACh was identified in *C. sorokiniana* U2-9 by using a quantitative colorimetric/fluorometric test. As shown in Figure 5, with the addition of 0.025 to 0.5 μg L^-1^ doses of AChE (acetylcholine inhibitor) to the ACh-pretreated samples, the dry weight and the lipid content were decreased by 12 to 50% and 15 to 45%, respectively. The effectiveness of ACh on ALA production was also neutralized by a 0.5 μg L^-1^ dosage of AChE (Table 3). The obtained results probably support the idea that endogenous ACh could have functions to improve biomass, lipids, and ALA contents of microalgae.

**Table 3:** **AChE neutralized ACh stimulatory action on ALA production**

			**Doses of AChE** **(μg L** ^**−1**^ **)**		
**FAS (%)**	**0**	**ACh**	**0.025**	**0.1**	**0.5**
C14:0	6.5	3.9	4.1	4.5	6.4
C16:0	33.2	22.5	24.3	26.0	29.7
C16:1	8.0	8.8	8.6	9.2	8.8
C16:2	3.2	5	5.1	4.7	3.8
C16:3	4.2	11.6	11.7	10.8	5.8
C18:0	10	2.0	2.7	3.0	7.8
C18:1	4.1	1.6	-	-	4.7
C18:2	16.2	18.2	18.6	17.9	16.2
C18:3	14.2	26.2	24.6	23.6	16.6

Note: ACh influence on production of ALA was reduced using AChE as an ACh inhibitor. ACh was applied at 5 μg L^-1^ at the initial phase of algal growth (data are presented as mean of three replicates).

### Generalization of ACh effects among species of the *Chlorella* genus

*Chlorella kessleri* UTEX 263*, Chlorella vulgaris* UTEX 395*,* and *Chlorella protothecoides* UTEX 256 were used to identify whether ACh has a general effectiveness through the *Chlorella* genus. As shown in Figure 1, ACh treatments contributed to higher biomass yields and higher total lipid contents by 20.6 to 43.7% and 15.5 to 19.0% over controls, respectively. The ALA contents in *C. protothecoides* UTEX 256 and *C. vulgaris* UTEX 395 were also improved by ACh up to 30.5% and 27.43%, respectively (Additional file 1: Table S-5).

### Effectiveness of ACh precursor and analogs on *C. sorokiniana* U2-9

Since we found that ACh had a broad effectiveness on different *Chlorella* species, we attempted to analyze whether the generality exists in the ACh precursor and analogs too. For this purpose, the precursor choline (CHOL) and four analogs (choline chloride (CHCL), choline hydroxide (CH), citicoline (INN) and phosphatidylcholine (PC)) were selected for the study.

The dry weight and the total lipid content were improved by all doses of ACh precursor and analogs, and our data uncovered the following order in the efficiency of ACh precursor and analogs: CHOL > PC > CHCL > CH > INN. The maximal increases in the dry weight, the total lipid content, and the lipid productivity were up to 5.17 g L^-1^, 354 mg g^-1^ of dry weight, and 182 mg L^-1^ day^-1^, respectively, by a 200 μg L^-1^ dosage of choline. We noticed that the ACh precursor and analog concentrations (up to 200 μg L^−1^) deployed were higher than that of ACh (5 μg L^-1^), and this dissimilarity might be due to differences in their modes of action. ACh might function as a regulator just as it works as a neurotransmitter in neurons; therefore, low concentrations could be sufficient to initiate a response. However, its precursor and analogs, for example, choline chloride, might function as a nutrient to fulfill the membrane development, and therefore higher concentrations would be needed.

The analysis of the fatty acid composition with ACh treatments indicated that the highest amount of ALA (31.08%) was achieved with a 200 μg L^-1^ dosage of CHOL; conversely, the lowest ALA production was obtained by INN (ALA production was decreased by 75.30% with a 200 μg L^-1^ dosage of INN).

The biodiesel yield was improved by the ACh precursor and analogs, although CHOL and CHCL were more efficient (Figure 2), and the IV and the CFPP were enhanced by ACh precursors and analogs except that the IV was reduced by 14 to 44% after using INN.

The biodiesel quality was also modified by some of the ACh precursors and analogs. As shown in Additional file 1: Table S-6, choline treatments obviously reduced the CN but also increased the IV of biodiesel. In contrast, citicoline treatments generated reversed effects on biodiesel. Changes in the ALA levels of the fatty acid profiles probably explain their differential effects, since the biodiesel quality is negatively determined by the percentage of polyunsaturated fatty acids (including ALA).

Choline and its derivatives have been identified in several microalgal species or have shown functions to microalgal physiology [26,27]. For example, 0.001 to 0.32% of the dry weight of microalgal species (for example, *Chlorophyta*, *Rhodophyta, Phaeophyta,* and *Euglenophyta*) is composed of choline. Synthetic choline derivatives such as choline chloride and (2-chloroethyl) trimethylammonium chloride are potent in increasing lipid contents of microalgal species [26,27]. Our current research distinguished two kinds of modes of action for choline and its derivatives. Briefly, they have achieved similar effects on the accumulation of total lipids as well as ALA but at different dose levels. The effective doses of acetylcholine are at least tenfold lower than those of choline and its other derivatives. We hypothesized that acetylcholine might function as a signaling molecule, while choline and its other derivatives function as nutrient molecules. More work is required to elucidate the underlying mechanisms.

In recent years, many researchers have attempted to improve microalgal lipid yields using different techniques including genetic engineering of important enzymes (such as acetyl-CoA carboxylase) of the lipid metabolism. Reports from the National Renewable Energy Laboratory in the USA indicate that the lipid contents of engineered microalgae were increased up to 60% and 40%, respectively, in laboratory and outdoor cultivations [28,29]. Besides the genetic techniques, the application of small bioactive molecules (such as ACh) offers an additional approach for the manipulation of microalgal biomass, lipid content, and fatty acid composition, as confirmed by this study.

The significance of the chemical approach based on small bioactive molecules might be summarized into three aspects. First, the small compounds offer a flexible technique which is easy to use and easy to integrate into the current production system of microalgae. Second, the small compounds promote the accumulation of both total lipids and high-value polyunsaturated fatty acids including ALA which could in turn reduce the costs of the microalgal production system, if the current methods to improve lipid yields are considered costly or time-consuming [30-32]. Third and more interestingly, the increased ALA yields with small compounds might offer a driving force to scale up biodiesel production from microalgae.

We preliminarily estimated the cost and profit of an optimized algal production system by ACh and its analogs which apparently supports the hypothesis of the driving force. The price of 250 mg ALA in the current market is 60 US dollars ($), while ACh and its cheap analogs (such as choline chloride) are at prices of 0.2 to 17.2 $ per 10 mg. When ACh and its analogs are used at 1 or 200 μg L^-1^ in a one-liter culture system for 10 days to generate a net increase in ALA yield by 140 mg, the profit reaches 34 $ (ALA) at a cost of 0.02 $ (small compounds). After ALA is separated, the rest of the fatty acids are transesterified into biodiesel with improved quality. The separation strategy should be optimized in future work. With everything taken together, ALA as a driving force to scale up biodiesel production might be considered.

## Conclusions

Based on our findings, we propose that non-neuronal ACh has a stimulatory role in growth and lipid accumulation of *Chlorella* species, and the exogenous usage of ACh at miniature doses promotes yields of biomass, lipids, and ALA. ACh and its precursor and analogs can be used as enhancers in a photoautotrophic microalgal production system for simultaneous production of ALA as a high-value fatty acid and lipid feedstock for biodiesel production. Moreover, microalgal ALA could drive biodiesel production economically and could improve biodiesel quality by the separation of ALA from the fatty acid mixture.

## Methods

### Microalgal strain, growth conditions and acetylcholine doses

*Chlorella sorokiniana* U2-9 belonging to the phylum Chlorophyta was chosen as a standard microalga in our study. The microalgae were maintained in 100 mL of Tris-Acetate-Phosphate (TAP) medium in 250-mL Erlenmeyer flasks under a 14-hour light [4800 (lux)]/10-hour dark cycle; the temperature was adjusted to 27°C, and the inoculation density was arranged on OD_680_ = 0.042.

ACh was purchased from the Sigma Aldrich company (≥99%), and it was dissolved in deionized water and then added to the medium by a syringe filter with 0.2-μm pore size at three different algal growth stages (initial, exponential, and stationary) at 0.125, 0.25, 0.5, 1, 5, and 10 (μg L^-1^) doses. The growth of algae was monitored by the optical density of the cultures at 680 nm, and OD_680_ values of 1.62 ± 0.15 and 3.15 ± 0.1 were obtained for exponential and stationary phases of algal growth, respectively.

### Detection of endogenous acetylcholine and usage of acetylcholinesterase

An EnzyChrom acetylcholine assay kit (EACL-100) was used for identification and quantification of ACh in *C. sorokiniana* U2-9, and the samples were analyzed using a GloMax*-*Multi Detection System (Promega). To estimate the actual influence of ACh, 0.025, 0.1, and 0.5 μg L^-1^ doses of acetylcholinesterase (AChE, as an ACh inhibitor, with activity ≥ 200 unit g^-1^) was used with a 5 μg L^-1^ dosage of ACh.

### Assessment of acetylcholine effectiveness at varied nutrition levels of media

Sets of experiments were conducted by altered levels of Tris acetate (TA) and phosphate buffer (PB) when a 5 μg L^-1^ dosage of ACh was included in the media to evaluate the stability of ACh at the altered culture conditions. In the first set of experiments, four levels of Tris acetate buffer with a 5 μg L^-1^ dosage of ACh were used (A:1.2 g L^-1^ Tris base and 5 mL L^-1^ glacial acetic acid, B: 2.4 g L^-1^ Tris base and 10 mL L^-1^ glacial acetic acid, C: 3.6 g L^-1^ Tris base and 12.5 mL L^-1^ glacial acetic acid, D: 4.8 g L^-1^ Tris base and 15 mL L^-1^ glacial acetic acid). In the second set of experiments, three levels of phosphate buffer with a 5 μg L^-1^ dosage of ACh were used (A: Na_2_HPO_4_: 5.8 g L^-1^, KH_2_PO_4_: 3.63 g L^-1^, B: Na_2_HPO_4_: 11.62 g L^-1^, KH_2_PO_4_: 7.26 g L^-1^, C: Na_2_HPO_4_: 17.42 g L^-1^, KH_2_PO_4_: 10.89 g L^-1^).

### ACh effectiveness on other species of the *Chlorella* genus

A 5 μg L^-1^ dosage of ACh was deployed at the initial growth phase of *C. kessleri* UTEX 263*, C. vulgaris* UTEX 395*,* and *C. protothecoides* UTEX 256 to evaluate the effectiveness of ACh on other species of *Chlorella.*

### Usage of acetylcholine precursor and analogs

Choline (CHOL), choline chloride (CHCL), choline hydroxide (CH), citicoline (INN), and phosphatidylcholine (PC) were used in doses of 50, 150, and 200 μg L^-1^ in the TAP medium to analysis the efficiency of the ACh precursor and analogs on *C. sorokiniana* U2-9.

### Analysis of growth, biomass and total lipid contents of *C. sorokiniana*

The growth of *C. sorokiniana* U2-9 was monitored by optical density (OD_680_) with a spectrophotometer device (T80 UV/VIS spectrometer), and the dry weight (g L^-1^) and the total lipid content (mg g^-1^ of dry weight) were obtained at three days past the stationary phase. The lipid productivity (mg L^-1^ day^-1^) was calculated using the following formula:

$$ \mathrm{Lipid}\ \mathrm{productivity} = \mathrm{C}\mathrm{L}/\mathrm{t} $$

where CL is the concentration of lipids (mg L^-1^) at the end of the culture and t is the duration of the culture (day).

### Extraction of lipids and transesterification

The obtained biomass from stationary phase was placed in 50-mL centrifuge tubes, 4 mL distilled water and 5 mL hydrochloridric acid (HCl) were added, and the samples were heated at 70°C in a water bath for 20 minutes. Afterward, 5 mL of ethanol was added, and the samples were cooled naturally at room temperature. Then 10 mL diethyl ether was added, and the samples were shaken and centrifuged (4000 rpm) for 1 and 2 minutes, and the ether layer was gathered into round flask; the process was repeated three times. Lastly, the total lipid content was obtained by evaporation of ether using a rotary evaporator. At the transesterification step, the obtained lipid was dissolved in chloroform and transferred into a 1.5-mL glass vial. Subsequently 1 mL 1 M sulphuric acid-methanol was added to the sample, and it was maintained for 1 hour at a temperature of 100°C. The samples were cooled naturally, and 500 μL distilled water was added and mixed by shaking for 2 minutes. Finally the samples were extracted with n-hexane three times, the organic phases were gathered and dried under nitrogen gas, and the obtained methyl ester was weighed.

### Fatty acid profiling analysis

A gas chromatography (GC) unit consisting of a FID detector (Agilent 7890) and a DB-WAX column (30 m × 0.32 mm × 0.50 μm) was used to determine the fatty acid content and composition of the samples. Methyl undecanoate was used as an internal standard and the analysis program was set as follows:

The temperature program comprised three phases; initially the temperature was increased from 50°C to 150°C at a rate of 10°C per minute, and held for 2 minutes; then the temperature was increased to 200°C from 150°C at a rate of 10°C per minute, and held for 6 minutes; and finally the temperature was increased to 230°C from 200°C at a rate of 10°C per minute, and held for 5 minutes. Carrier gas (N_2_) velocity: 3 mL per minute. Detector: hydrogen flame detector, the velocity of H_2_ was 30 mL per minute, and the velocity of air was 300 mL per minute. The detector temperature and injector temperature were adjusted to 300 and 280°C, respectively.

### Estimation of biodiesel properties by using fatty acid profiles

The biodiesel yield and parameters of biodiesel quality were estimated by the molecular structure of fatty acids using a set of formulas described by Nascimento and colleagues [25].

1. Biodiesel yield = fatty acid methyl ester/algae biomass* lipid content
2. Saponification (SV) and Iodine value (IV)
  $$ \mathrm{S}\mathrm{V} = \Sigma \left(560*\mathrm{N}\right)/\mathrm{M}\ \mathrm{I}\mathrm{V} = \Sigma \left(254*\mathrm{D}\mathrm{N}\right)/\mathrm{M} $$

where D is number of double bonds, M is fatty acid (FA) molecular mass, and N is the percent of each FA.

(3) Cetane number (CN) was calculated based on SV and IV.

$$ \mathrm{C}\mathrm{N} = 46.3 + \left(5458/\mathrm{S}\mathrm{V}\right)\hbox{-} \left(0.225*\mathrm{I}\mathrm{V}\right) $$

Degree of unsaturation (DU)

(4) DU = MUFA+ (2*PUFA)

where MUFA is monounsaturated fatty acid and PUFA is polyunsaturated fatty acid.

(5) Long chain saturation factor (LCSF)

LCSF = (0.1*C16) + (0.5*C18) + (1*C20) + (1.5*C22) + (2*C24)

where C16, C18, C20, C22, C24 are weight percentage of each fatty acid.

(6) Cold filter plugging point (CFPP) calculated based on LCSF

CFPP = (3.1417*LCSF)-16.477.

## Additional file

Additional file 1:
**Supplementary supporting data.** Table S-1. The effects of ACh on growth and lipid accumulation in *C. sorokiniana U2-9 at different growth stages.* Table S-2. Fatty acid profiles and estimated properties of biodiesel of *C. sorokiniana U2-9 under ACh doses at different growth stages.* Table S-3. Fatty acid profiles and estimated properties of biodiesel at different levels of Tris base and acetic acids. Table S-4. Fatty acid profiles and estimated properties of biodiesel at different phosphate buffer levels. Table S-5. Profiles of fatty acids and estimated properties of biodiesel from different species of the *Chlorella* genus. Table S-6. Comparative fatty acid profiles (%) and estimated biodiesel properties affected by precursor and analogs of acetylcholine at different doses.

## Abbreviations

ACh: Acetylcholine
ALA: Alpha-linolenic acid
CFPP: Cold filter plugging point
CH: Choline hydroxide
CHCL: Choline chloride
CHOL: Choline
CN: Cetane number
DU: Degree of unsaturation
INN: Citicoline
IV: Iodine value
LCSF: Long-chain saturation factor
PC: Phosphatidylcholine
SV: Saponification
