# Supplementary material for: Simultaneous improvement in production of microalgal biodiesel and high-value alpha-linolenic acid by a single regulator acetylcholine
Source: Biotechnol Biofuels. 2015 Jan 29;8:11. doi: 10.1186/s13068-015-0196-0 (PMC4329656; doi:10.1186/s13068-015-0196-0)
Supplement: Additional file 1: — Supplementary supporting data. Table S-1. The effects of ACh on growth and lipid accumulation in C. sorokiniana U2-9 at different growth stages. Table S-2. Fatty acid profiles and estimated properties of biodiesel of C. sorokiniana U2-9 under ACh doses at different growth stages. Table S-3. Fatty acid profiles and estimated properties of biodiesel at different levels of Tris base and acetic acids. Table S-4. Fatty acid profiles and estimated properties of biodiesel at different phosphate buffer levels. Table S-5. Profiles of fatty acids and estimated properties of biodiesel from different species of the Chlorella genus. Table S-6. Comparative fatty acid profiles (%) and estimated biodiesel properties affected by precursor and analogs of acetylcholine at different doses. [file 13068_2015_196_MOESM1_ESM.docx]

**Table S-1. The effects of ACh on growth and lipid accumulation in C. sorokiniana U2-9 at different growth stages**

| **ACh** (µg L^-1^) | | | | | | | | | | | | | |  |  |
| --- | --- | --- | --- | --- | --- | --- | --- | --- | --- | --- | --- | --- | --- | --- | --- |
| **0** | | **0.125** | | **0.25** | | **0.5** | | **1** | | **5** | | **10** | |  |  |
| **Initial phase** | Dry weight (gL^-1^) |  | 2.16±0.2 | | 2.2±2 | | 2.2±0.2 | | 2.4±0.17 | | 2.43±0.2 | | 2.73±0.05 | | 2.66±0.15 |
|  | Total lipid content (mg g^-1^ of dry weight) |  | 214.3±14 | | 223.3±16 | | 231.6±12 | | 252±10 | | 296±26 | | 303±24 | | 190±12 |
|  | Lipid productivity  (mgL^-1^day^-1^) |  | 46.4±6 | | 49.3±7 | | 50.8±4 | | 60.5±4 | | 72.4±8 | | 82.8±6 | | 50.7±5 |
| **Exponential phase** | Dry weight (gL^-1^) |  | 2.16±0.2 | | 2.36±0.2 | | 2.5±0.1 | | 2.7±0.1 | | 2.7±0.1 | | 2.9±0.1 | | 3.2±0.15 |
|  | Total lipid content (mg g^-1^ of dry weight) |  | 214.3±24 | | 233.3±18 | | 252.3±26 | | 278±22 | | 309.3±31 | | 312±54 | | 163.3±21 |
|  | Lipid productivity  (mgL^-1^day^-1^) |  | 46.4±6 | | 55.4±5 | | 63.6±7 | | 75.3±5 | | 83.6±6 | | 92.7±10 | | 52.4±8 |
| **Stationary phase** | Dry weight (gL^-1^) |  | 2.06±0.2 | | 2.03±0.2 | | 2.01±0.1 | | 2.02±0.2 | | 2.03±0.1 | | 2.02±0.1 | | 2.02±0.08 |
|  | Total lipid content (mg g^-1^ of dry weight) |  | 206.7±17 | | 207.8±14 | | 207±16 | | 205.4±12 | | 208.3±14 | | 204.8±16 | | 208.6±10 |
|  | Lipid productivity  (mgL^-1^day^-1^) |  | 42.6±7 | | 41.8±5 | | 42.7±4 | | 42.5±3 | | 42.7±6 | | 42.5±7 | | 42.4±5 |

**Table S-2. Fatty acid profiles and estimated properties of biodiesel of C. sorokiniana U2-9 under ACh doses at different**

|  | **ACh Doses** (µg L^-1^) | | | | | | | | | | | | | | | | | | | | | |
| --- | --- | --- | --- | --- | --- | --- | --- | --- | --- | --- | --- | --- | --- | --- | --- | --- | --- | --- | --- | --- | --- | --- |
|  | **Initial phase** | | | | | | | **Exponential phase** | | | | | | | **Stationary phase** | | | | | | | |
| **FAS (%)** | **0** | **0.125** | **0.25** | **0.5** | **1** | **5** | **10** | **0** | **0.125** | **0.25** | **0.5** | **1** | **5** | **10** | **0** | **0.125** | **0.25** | **0.5** | **1** | **5** | **10** |  |
| C14:0 | 3.1 | 4.4 | 3.8 | 2.6 | 7.5 | 4 | 4.5 | 2.83 | 2.07 | 3.0 | 2.4 | 3.2 | 2.7 | 2.2 | 3.8 | 3.0 | 2.1 | 2.1 | - | 3.4 | 3.5 |  |
| C16:0 | 26.3 | 26.5 | 24.8 | 24.7 | 37.1 | 23.9 | 26.6 | 28.85 | 29.48 | 27.3 | 24.6 | 22.9 | 23.7 | 22.0 | 35.3 | 35.8 | 28.7 | 28.7 | 32.1 | 32.3 | 31.9 |  |
| C16:1 | 6.4 | 5.5 | 6.7 | 4.1 | - | 6.4 | 6 | 6.17 | 5.78 | 6.6 | 6.3 | 6.0 | 4.8 | 6.9 | 4.6 | 5.7 | 4.3 | 4.3 | 0 | 4.2 | 4.2 |  |
| C16:2 | 10.7 | 8.4 | 8.8 | 8.5 | 7 | 8.5 | 7 | 7.98 | 7.55 | 7.2 | 9.3 | 9.5 | 9.1 | 10.2 | 6.8 | 6.3 | 9.7 | 9.7 | 7.9 | 7.2 | 7.4 |  |
| C16:3 | 5.1 | 7.2 | 7.6 | 6.9 | 5.2 | 8.5 | 6.7 | 6.39 | 6.60 | 6.9 | 8.0 | 9.0 | 8.7 | 10.3 | 6.0 | 4.5 | 6.5 | 6.5 | 6.8 | 6.3 | 6.1 |  |
| C18:0 | - | - | - | - | - | - | - | 2.64 | 4.33 | 2.8 | 2.6 | 1.8 | 2.7 | 3.1 | 9.7 | 6.9 | 5.4 | 5.4 | 6.8 | 4.1 | 6.9 |  |
| C18:1 | 11.9 | 6.9 | 6.7 | 6.6 | - | 9.0 | 7.8 | 9.25 | 8.44 | 9.8 | 7.9 | 8.9 | 8.0 | 7.5 | - | - | - | - | - | - | - |  |
| C18:2 | 21.8 | 19 | 19.8 | 22.9 | 19.6 | 18.0 | 18.5 | 18.53 | 17.55 | 17.1 | 19.5 | 18.8 | 20.1 | 18.5 | 14.7 | 17.8 | 21.4 | 21.4 | 19.9 | 20.9 | 19.3 |  |
| C18:3 | 14.7 | 22 | 21.8 | 23.6 | 23.7 | 21.6 | 22.8 | 17.3 | 18.16 | 18.8 | 19.0 | 19.6 | 20.0 | 18.8 | 18.7 | 19.6 | 21.5 | 21.5 | 26.2 | 21.2 | 20.9 |  |
| SV | 210.9 | 211.3 | 211.4 | 209.8 | 213.3 | 211.1 | 211.1 | 210.9 | 210.5 | 210.8 | 210.8 | 210.1 | 210.5 | 210.9 | 211.9 | 211.5 | 211.3 | 210.8 | 209.2 | 211.5 | 212.0 |  |
| IV | 133.8 | 145.1 | 149.1 | 154.2 | 130.2 | 149.1 | 143.3 | 131.0 | 130.1 | 133.9 | 144.2 | 148.5 | 148.0 | 150.9 | 114.9 | 118.5 | 142.7 | 141.6 | 144.8 | 134.2 | 130.1 |  |
| DU | 122.9 | 125.6 | 129.4 | 134.5 | 111.0 | 128.6 | 123.8 | 115.9 | 115.9 | 117.0 | 126.2 | 129.0 | 128.8 | 130.4 | 97.5 | 102.6 | 120.5 | 122.9 | 122.1 | 115.8 | 111.8 |  |
| LCSF | 2.6 | 2.6 | 2.5 | 2.5 | 3.7 | 2.4 | 2.6 | 4.2 | 5.11 | 4.1 | 3.7 | 3.2 | 3.7 | 3.7 | 8.4 | 7.05 | 5.5 | 5.6 | 6.6 | 5.28 | 6.6 |  |
| CFPP(ºC) | -8.1 | -8.1 | -8.7 | -8.7 | -4.8 | -9.0 | -8.1 | -3.3 | -0.4 | -3.4 | -4.6 | -6.3 | -4.6 | -4.6 | 9.9 | 5.6 | 0.9 | 1.0 | 4.3 | 0.1 | 4.4 |  |
| CN | 42.1 | 39.5 | 38.6 | 37.6 | 42.6 | 38.6 | 39.9 | 42.7 | 42.9 | 42.0 | 39.7 | 38.7 | 38.9 | 38.2 | 46.2 | 45.4 | 40.0 | 40.3 | 39.7 | 41.9 | 42.7 |  |

**growth stages**

Note: Acetylcholine was able to increase the alpha-linolenic acid (C18:3) at different growth stages of C. sorokiniana U2-9 in a range from 14% to 60%. Moreover, the estimation of biodiesel properties described, the iodine value was increased and cold filter plug in point was decreased by changes in fatty acid composition in ACh-treated samples. Data are presented as the mean of three replicates.

**Table S-3. Fatty acid profiles and estimated properties of biodiesel at**

**different Tris acetate levels**

| **- ACh** | | | | **+ACh** | | | |  |
| --- | --- | --- | --- | --- | --- | --- | --- | --- |
| **FAS (%)** | A | B | C | D | A | B | C | D |
| C14:0 | 2.7 | 2.0 | 2.6 | 2.8 | 3.3 | 2.2 | 2.4 | 2.1 |
| C16:0 | 32.6 | 27.7 | 30.2 | 30.5 | 35.6 | 27.8 | 28.8 | 28.3 |
| C16:1 | 2.8 | 2.9 | 3.3 | 3.4 | 4.1 | 3.1 | 3.3 | 2.6 |
| C16:2 | 5.3 | 6.4 | 4.4 | 5.6 | 3.7 | 5.1 | 5.4 | 6.1 |
| C16:3 | 3.0 | 4.7 | 5.4 | 3.6 | 3.9 | 5.5 | 5.7 | 5.2 |
| C18:0 | 4.6 | 3.7 | 4.2 | 3.7 | 5.5 | 3.7 | 3.7 | 3.9 |
| C18:1 | 10.1 | 8.8 | 9.5 | 9.3 | 9.9 | 8.3 | 7.8 | 9.6 |
| C18:2 | 26.2 | 26.0 | 21.4 | 25.5 | 18.7 | 23.5 | 22.6 | 25.7 |
| C18:3 | 12.4 | 17.3 | 18.8 | 15.2 | 15.0 | 20.6 | 20.1 | 16.2 |
| SV | 210.2 | 208.8 | 209.4 | 209.4 | 209.3 | 208.9 | 209.4 | 208.9 |
| IV | 107.5 | 132.9 | 127.7 | 122.2 | 113.2 | 136.7 | 134.6 | 130.4 |
| DU | 96.8 | 120.9 | 113.1 | 112.9 | 106.8 | 121.0 | 118.8 | 118.9 |
| LCSF | 6.3 | 4.6 | 5.1 | 4.9 | 5.5 | 4.6 | 4.7 | 4.7 |
| CFPP(ºC) | 3.4 | 1.8 | -0.4 | 1.0 | 1.0 | 1.9 | -1.5 | 1.4 |
| CN | 48.0 | 42.5 | 43.6 | 44.8 | 46.9 | 41.6 | 42.0 | 43.0 |

Note: A: 1.2 g L^-1^ Tris base and 5 mL L^-1^ glacial acetic acid, B: 2.4 g L^-1^ Tris base and 10 mL L^-1^ glacial acetic acid, C: 3.6 g L^-1^ Tris base and 12.5 mL L^-1^ glacial acetic acid, D: 4.8 g L^-1^ Tris base and 15 mL L^-1^ glacial acetic acid. Data are presented as the mean of three replicates.

**Table S-4. Fatty acid profiles and estimated properties**

**of biodiesel at different phosphate buffer levels.**

| - ACh | | | +ACh | | |  |
| --- | --- | --- | --- | --- | --- | --- |
| **FAS (%)** | **A** | **B** | **C** | **A** | **B** | **C** |
| C14:0 | 2.7 | 4.4 | 3.1 | 2.4 | 1.8 | 3.1 |
| C16:0 | 25.5 | 28.7 | 27.3 | 23.2 | 23.7 | 27.6 |
| C16:1 | 4.8 | 4.4 | 4.4 | 5.0 | 4.1 | 3.9 |
| C16:2 | 7.6 | 2.6 | 6.1 | 7.7 | 7.4 | 2.9 |
| C16:3 | 11.4 | 0.8 | 7.6 | 12.6 | 11.3 | 0.5 |
| C18:0 | - | 2.7 | 2.8 | - | 1.9 | 2.5 |
| C18:1 | - | 17.3 | 6.2 | - | 4.1 | 18.9 |
| C18:2 | 19.9 | 16.7 | 20.5 | 19.0 | 19.2 | 17.2 |
| C18:3 | 27.9 | 22.1 | 21.8 | 29.8 | 26.0 | 23.2 |
| SV | 211.4 | 208.6 | 210.3 | 211.2 | 210.3 | 207.7 |
| IV | 167.3 | 118.6 | 142.5 | 175.2 | 163.7 | 123.1 |
| DU | 138.6 | 106.3 | 122.9 | 143.6 | 136.6 | 110.6 |
| LCSF | 2.5 | 4.2 | 4.1 | 2.3 | 3.2 | 4.0 |
| CFPP(ºC) | -8.4 | -3.1 | -3.5 | -9.2 | -6.0 | -3.8 |
| CN | 32.7 | 44.8 | 40.1 | 34.4 | 35.4 | 45.7 |

Note: The amount of ALA (C18:3) was increased using ACh under different levels of the phosphate buffer (PB). A-C describes the applied levels of the PB, A: Na_2_HPO_4_ 5.8 g L^-1^, KH_2_PO_4_ 3.63 g L^-1^, B: Na_2_HPO_4_ 11.62 g L^-1^, KH_2_PO_4_ 7.26 g L^-1^, C: Na_2_HPO_4_ 17.42 g L^-1^, KH_2_PO_4_ 10.89 g L^-1^. Data are presented as the mean of three replicates.

**Table S-5. Profiles of fatty acids and estimated properties of biodiesel from different species of the *Chlorella* genus.**

| **- ACh** | | | **+ACh** | | |  |
| --- | --- | --- | --- | --- | --- | --- |
| **FAs (%)** | *C. Protothecoides* | *C. Vulgaris* | *C. Kessleri* | *C. Protothecoides* | *C. Vulgaris* | *C. Kessleri* |
| C14:0 | 4.1 | 4.3 | 3.7 | 3 | 4.0 | 4.1 |
| C16:0 | 21.7 | 21.5 | 25.3 | 25.7 | 26.2 | 28.1 |
| C16:1 | 5.7 | 6.0 | 5.5 | 1.8 | 6.1 | 4.2 |
| C16:2 | 7.0 | 7.1 | 4.8 | 4.5 | 4.8 | 3.5 |
| C16:3 | 8.2 | 8.0 | 4.4 | 5.6 | 7.2 | 2.6 |
| C18:0 | 1.9 | 2.1 | 2.6 | 1.9 | 1.3 | 2.2 |
| C18:1 | 8.1 | 8.1 | 11.2 | 8.5 | 6.2 | 14.8 |
| C18:2 | 19.0 | 18.7 | 18.4 | 18.2 | 16.4 | 16.3 |
| C18:3 | 24.1 | 23.9 | 23.8 | 30.5 | 27.4 | 24.1 |
| SV | 209.4 | 210.5 | 210.4 | 209.0 | 210.6 | 208.7 |
| IV | 137.4 | 151.5 | 152.2 | 127.8 | 148.2 | 152.1 |
| DU | 63.1 | 65.6 | 65.7 | 58.7 | 54.9 | 55.8 |
| LCSF | 3.8 | 3.2 | 3.1 | 3.9 | 3.3 | 3.5 |
| CFPP(ºC) | -4.4 | -6.4 | -6.5 | -4.2 | -6.1 | -5.3 |
| CN | 41.4 | 38.1 | 38.0 | 43.6 | 38.8 | 38.2 |

Note: ACh increased the amounts of alpha-linolenic acid (C18:3) in *C. protothecoides* UTEX 256 and *C. vulgaris* UTEX 395*.*

|  | **Control** | **CHOL** | | | **CH** | | | **CHCL** | | | **INN** | | | **PC** | | |
| --- | --- | --- | --- | --- | --- | --- | --- | --- | --- | --- | --- | --- | --- | --- | --- | --- |
| **FAS (%)** | **-** | **50** | **150** | **200** | **50** | **150** | **200** | **50** | **150** | **200** | **50** | **150** | **200** | **50** | **150** | **200** |
| C14:0 | 5.2 | 2.9 | 3.2 | 3.3 | 3.6 | 3.5 | 3.2 | 2.7 | 2.9 | 3.1 | 3.9 | 4.7 | 5.6 | 3.3 | 2.9 | 3.2 |
| C16:0 | 33.5 | 21.9 | 27.1 | 23.0 | 36.4 | 36.8 | 43.8 | 24.6 | 26.3 | 22.2 | 39.9 | 44.7 | 58.1 | 28.0 | 23.3 | 26.7 |
| C16:1 | 7.2 | 5.2 | 6.3 | 6.2 | 6.0 | 6.6 | 6.5 | 4.5 | 5.3 | 4.8 | 5.1 | 7.1 | 10.1 | 4.6 | 4.4 | 6.5 |
| C16:2 | 3.7 | 6.2 | 4.8 | 5.6 | 4.2 | 3.48 | 3.1 | 6.5 | 6.8 | 8.0 | 4.2 | 2.6 | 1.0 | 5.6 | 7.0 | 5.8 |
| C16:3 | 4.4 | 12.6 | 7.0 | 10.1 | 4.7 | 4.48 | 4.1 | 9.7 | 8.5 | 11.1 | 3.7 | 2.6 | 7.8 | 7.1 | 9.8 | 9.1 |
| C18:0 | 3.5 | 2.9 | 3.3 | 4.2 | 5.9 | 3.08 | 7.2 | 3.9 | 4.2 | 2.9 | 6.7 | 9.0 | - | 4.0 | 3.7 | - |
| C18:1 | 7.7 | 4.1 | 4.0 | 2.8 | 4.2 | 7.39 | - | 7.1 | 3.0 | 3.5 | 5.5 | 5.0 | 6.4 | 8.6 | 7.4 | 3.9 |
| C18:2 | 14.1 | 14.3 | 14.7 | 13.3 | 14.3 | 14.8 | 14.1 | 15.1 | 16.9 | 17.7 | 15.2 | 11.0 | 5.6 | 14.6 | 16.0 | 14.5 |
| C18:3 | 20.5 | 29.5 | 29.6 | 31.0 | 20.4 | 19.0 | 17.8 | 25.6 | 25.8 | 26.3 | 15.6 | 13.0 | 5.1 | 23.4 | 25.4 | 30.1 |
| SV | 211.6 | 210.8 | 210.4 | 210.6 | 211.4 | 210.6 | 212.3 | 210.3 | 210.6 | 210.8 | 211.6 | 212.6 | 217.2 | 209.2 | 210.2 | 211.2 |
| IV | 116.9 | 166.8 | 148.5 | 160.2 | 114.4 | 151.3 | 99.5 | 151.3 | 148.9 | 162.5 | 100.1 | 80.4 | 65.8 | 135.8 | 153.5 | 158.3 |
| DU | 100.6 | 135.0 | 122.5 | 119.0 | 56.6 | 95.6 | 68.9 | 95.6 | 90.6 | 99.5 | 57.7 | 48.7 | 44.5 | 85.6 | 96.4 | 100.6 |
| LCSF | 5.1 | 3.6 | 4.3 | 4.4 | 6.6 | 4.4 | 7.9 | 4.4 | 4.7 | 3.6 | 7.3 | 8.9 | 5.8 | 4.8 | 4.2 | 2.6 |
| CFPP(ºC) | -0.4 | -5.0 | -2.7 | -2.5 | 4.3 | -2.6 | 8.6 | -2.6 | -1.5 | -4.9 | 6.5 | 11.7 | 1.8 | -1.4 | -3.2 | -8.1 |
| **CN** | 45.7 | 34.6 | 38.8 | 36.1 | 46.3 | 38.2 | 49.6 | 38.2 | 38.7 | 35.6 | 49.5 | 53.8 | 56.6 | 41.8 | 37.7 | 36.5 |

**Table S-6. Comparative fatty acid profiles (%) and estimated biodiesel properties affected by precursor and analogs of acetylcholine at different doses (µg L^-1^).**

Note: The relative amount of ALA (C18:3) in C. sorokiniana U2-9 was increased by usage of CHOL, CHCL and PC. CHOL= Choline, CHCL = Choline chloride, CH= Choline hydroxide, INN= Citicoline, PC= Phosphatidylcholine. Data are presented as the mean of three replicates.
